# New Paradigms in Antithrombotic Strategies: A Leap into the Future of Cardiovascular Medicine

**DOI:** 10.3390/jcm11102693

**Published:** 2022-05-10

**Authors:** Giulio Francesco Romiti

**Affiliations:** Department of Translational and Precision Medicine, Sapienza—University of Rome, 00161 Rome, Italy; giuliofrancesco.romiti@uniroma1.it; Tel.: +39-06-4997-0425

During the last decades, significant improvements have changed the landscape of antithrombotic treatment strategies and, more generally, the treatment of thrombotic and cardiovascular diseases. Among these, atrial fibrillation (AF), venous thromboembolism (VTE) and ischemic heart disease (IHD) have seen major developments, which have also been reflected by guidelines and changes in clinical practice [1,2,3]. Since the introduction of nonvitamin K antagonist oral anticoagulants (NOACs), which represented the first large-scale paradigm shift in the treatment of thrombotic diseases, research has focused on the unmet need for safer and effective anticoagulation, especially in those patients who are at higher risk of thrombotic and haemorrhagic events. These patients, whose prevalence is steadily increasing due to the progressive aging of population, and the higher clinical complexity currently found in clinical practice [4], require a tailored and comprehensive approach to thromboembolic risk prevention.

The Special Issue “Current Advances and Future Directions for Antithrombotic Treatment Strategies” has been dedicated to collect contributions on current and future developments in the broad research area of thrombotic diseases, from AF and VTE to the thrombotic aspects related to the COVID-19 pandemic, which imposed a high burden of morbidity and mortality over the last few years.

Among the contributions, two were indeed focused on the recent COVID-19 pandemic. A systematic review and meta-analysis of 31 studies showed how new-onset AF represents a common complication in COVID-19 patients, being found in up to 8% of hospitalized patients, and also showed that AF is associated with a significant increase in the risk of all-cause mortality [5]. These results are in accordance with those found in other similar scenarios, such as sepsis [6,7,8] and community-acquired pneumonia [9], and offer an interesting outlook on the relationship between infections, inflammation, and cardiovascular disease, all being connected by thrombosis [10]. On the other hand, Protasiewicz et al. reported a retrospective analysis on the impact of anticoagulation before the SARS-CoV-2 infection on the clinical course and outcome of COVID-19; while being limited by low statistical power, the authors did not find any statistically significant difference according to the anticoagulation status [11]. This underlines the great uncertainty regarding the thrombotic aspects of COVID-19 and the optimal strategies to prevent the occurrence of thrombosis.

Aside from COVID-19, other contributions focused on some of the hot topics in the antithrombotic research area. Camelo-Castillo and colleagues provided a comprehensive outlook on the interplay between the gut microbiota and the quality of anticoagulation control during treatment with vitamin K antagonists (VKA) [12], which still represent the therapeutic choice for several medical conditions that require anticoagulation. The relationship between gut microbiota and cardiovascular disease has been one of the most exciting areas of research during the last years [13], although the complex interplays with treatments and response to drugs are still mostly unknown and will certainly represent a topic of prominent interest in the future.

In the review from Khatri et al., the authors summarise current developments in VTE prophylaxis in surgical patients [14], also with a specific focus on the role of mechanical prophylaxis. This topic, which has seen several changes in clinical practice since the introduction of NOACs, has significant clinical implications in everyday practice. VTE prevention landscape is constantly in the need of improvements in the risk–benefit profile of the preventive approaches implemented, especially in those patients at high risk of bleeding. Significant advances are expected from the new drugs that target factor XI, and further evidence will be hopefully available in the next few years [15].

Moving forward, several contributions focused on the real-world assessment of the efficacy and safety of NOACs. Crocetti and colleagues provided an Italian-based real-word confirmation of the overall superiority of NOACs over VKA for thromboembolic risk prevention in AF patients [16], in line with previous reports and with also interesting data for individual drugs. In the paper from Faggiano et al., the authors provided a retrospective analysis on the prevalence and clinical course of left atrial thrombus, also with insights on the role of anticoagulation and, specifically, of NOACs and VKA [17]. Real-world evidence on this topic is very much needed, given the uncertainty and the implications on clinical practice. Finally, Zerah and colleagues described treatment patterns for antithrombotic combinations using a nationwide French database and showed how a significant proportion of the combinations was inappropriately prescribed or noncompliant with international guideline recommendations, with an unsurprising impact on the risk of major bleeding during follow-up [18]. Data on these issues are crucial due to the growing number of patients with complex indications for antithrombotic treatments, the rise of multimorbidity, and the growing need for tailored approaches, especially for high-risk patients.

Many other open questions raise great interest in the broad research area of prevention and treatment of thrombotic diseases. Particularly, the dual-pathway inhibition, wherein anticoagulants and antiplatelets are used simultaneously [19], as well as the interplay between inflammation and thrombosis [10], and the discovery of new therapeutic targets and antithrombotic drugs [20] are some of the most exciting frontiers in this research area. These topics are posed to dramatically change the landscape and shape the future of cardiovascular diseases management during the next decades. The growing proportion of patients who are at both higher risk of thrombosis and bleeding drives research towards the quest for safer and effective antithrombotic strategies, without neglecting the need for a more comprehensive and integrated approach to the treatment and prevention of thrombosis.

## Data Availability

Not applicable.
